# Supplementary material for: Identification of Novel Pathways Associated with Patterned Cerebellar Purkinje Neuron Degeneration in Niemann-Pick Disease, Type C1
Source: Int J Mol Sci. 2019 Dec 31;21(1):292. doi: 10.3390/ijms21010292 (PMC6981888; doi:10.3390/ijms21010292)
Supplement: Supplementary file 1 [file ijms-21-00292-s001.zip › supplementary_all/supplementary_materials.pdf]

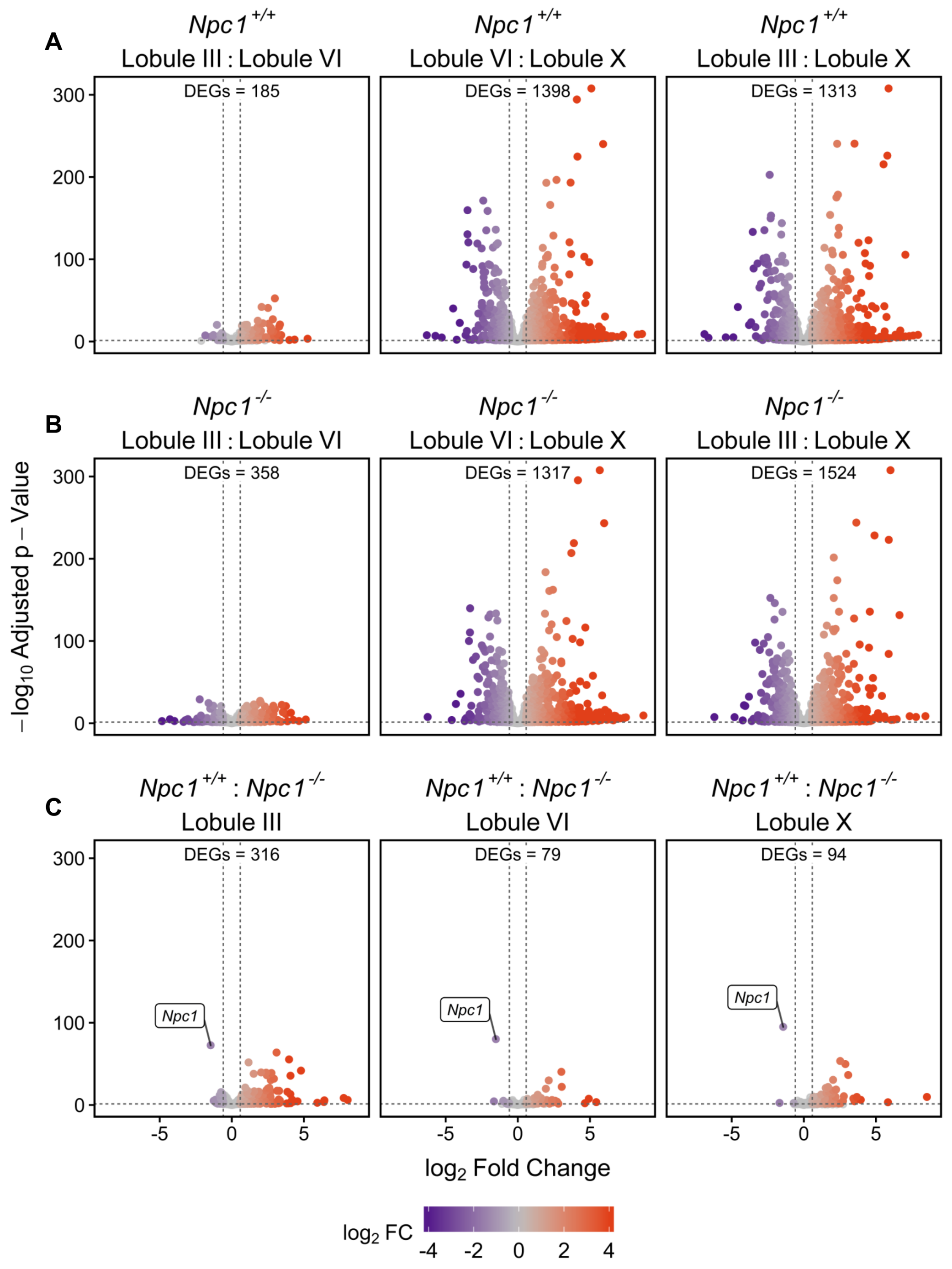

Supplemental Figure 1. Volcano plots of differential gene expression. (**A-C**) Volcano plots depicting the $\log_2$ fold change (x-axis) and the $-\log_{10}$ adjusted p-value (y-axis). The number of differentially expressed genes (DEGs), as defined as having an adjusted p-value < 0.05 and an absolute $\log_2$ fold change > 0.58 (which corresponds to a 1.5-fold change), is displayed for each comparison. DEGs are depicted as orange or purple, for upregulated or downregulated, respectively. Genes that are not differentially expressed are shown in gray. (**A**) Changes in gene expression within the *Npc1*$^{+/+}$ cerebellum, including the anterior comparison of lobule III to lobule VI (left), the anterior to nodular comparison of lobule VI to lobule X (center), and an additional anterior to nodular comparison of lobule III to lobule X (right). (**B**) Changes in gene expression within the *Npc1*$^{-/-}$ cerebellum, including the comparison of lobule III to VI (left), the comparison of lobule VI to X (center), and the comparison of lobule III to X (right). (**C**) Comparison of inter-genotypic gene expression changes between *Npc1*$^{+/+}$ and *Npc1*$^{-/-}$ in the cerebellar lobule III (left), lobule VI (center), and lobule X (right).

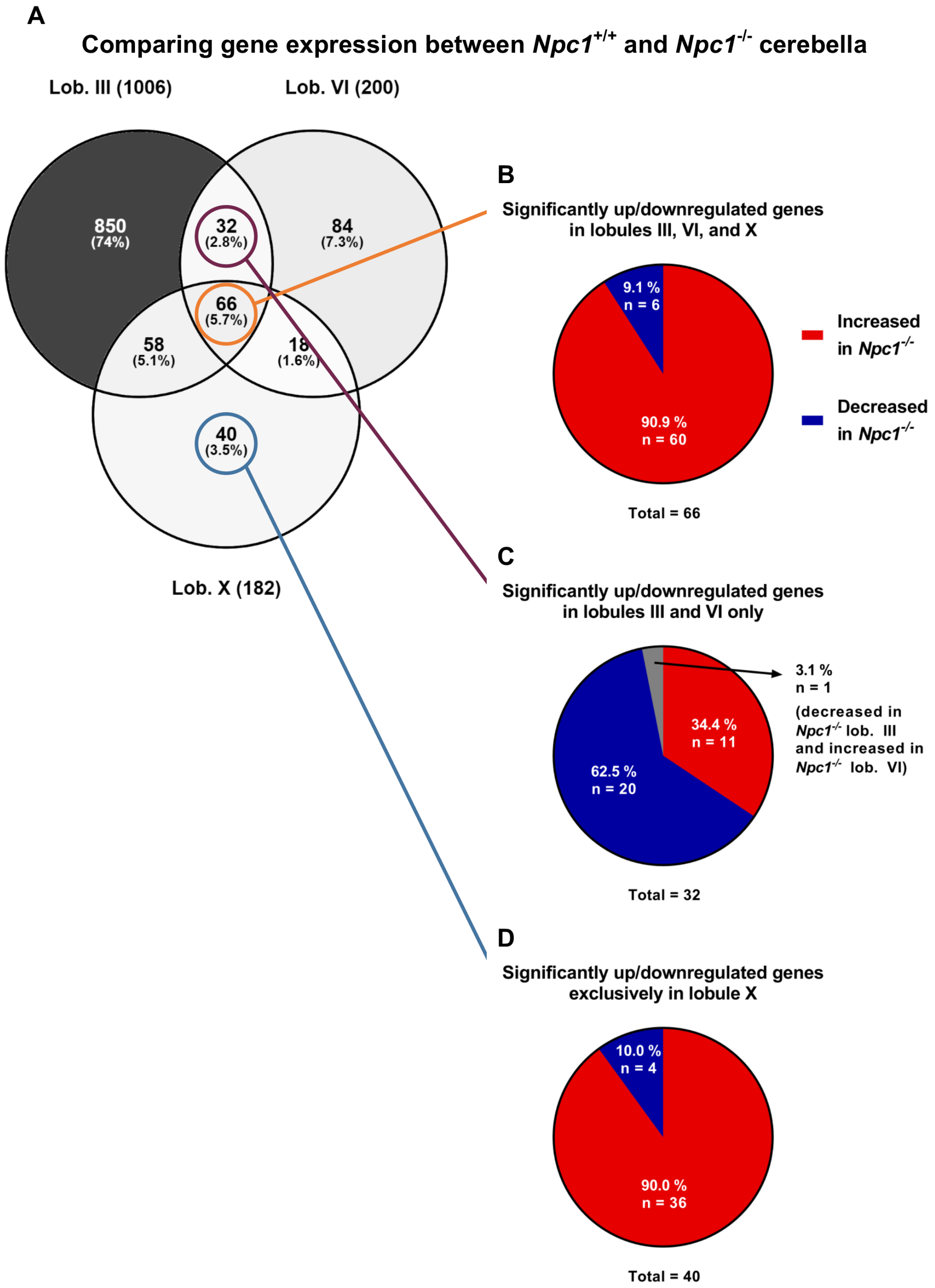

Supplemental Figure 2. Venn diagrams of differential gene expression comparing lobules between *Npc1*$^{+/+}$ and *Npc1*$^{-/-}$ samples. (**A**) Venn diagraming showing genes differentially expressed genes in each lobule of *Npc1*$^{-/-}$ compared to *Npc1*$^{+/+}$. (**B-D**) Number of significantly increased (red) and decreased (blue) genes in *Npc1*$^{-/-}$ for each comparison of interest.

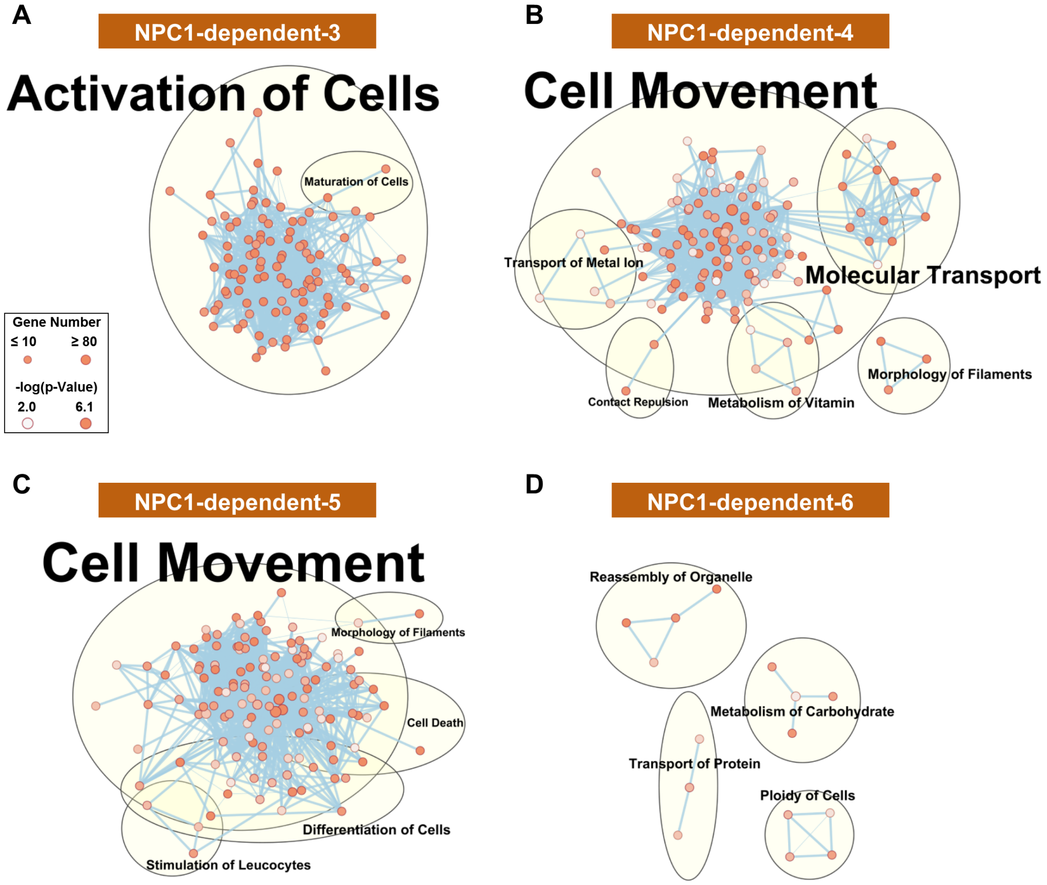

Supplemental Figure 3. Functional enrichment for additional disease-specific expression pattern modules. IPA functional analysis of the significantly enriched molecular and cellular functions for the additional disease-specific modules of NPC1-dependent-3 (**A**), NPC1-dependent-4 (**B**), NPC1-dependent-5 (**C**), and NPC1-dependent-6 (**D**). Each node represents a specific molecular and cellular function, the size of which corresponds to the number of genes included in each function. The edges represent the number of shared genes between each function.

A

IPA Canonical Pathways for WGCNA Module: NPC1-dependent-8

	Ingenuity Canonical Pathway	$-\log_{10}$ pvalue	Gene Count	Gene Ratio	Genes in Pathway
1	CDP-diacylglycerol Biosynthesis I	2.77	4	0.167	*Lpcat2, Cds1, Lpcat4, Mboat1*
2	Phosphatidylglycerol Biosynthesis II (Non-plastidic)	2.63	4	0.154	*Lpcat2, Cds1, Lpcat4, Mboat1*
3	Branched-chain α-keto acid Dehydrogenase Complex	2.55	2	0.500	*Dbt, Bckdhb*
4	Triacylglycerol Biosynthesis	2.49	5	0.109	*Lpcat2, Lpcat4, Mboat1, Dbt, Plpp2*
5	Acetyl-CoA Biosynthesis I (Pyruvate Dehydrogenase Complex)	2.03	2	0.286	*Pdha1, Dbt*
6	Small Cell Lung Cancer Signaling	1.96	6	0.071	*Pias3, Traf4, Abl1, Fgfr2, Cycs, Traf1*
7	Reelin Signaling in Neurons	1.80	6	0.065	*Map3k9, Mapk10, Fgfr2, Arhgef9, Arhgef10, App*
8	Calcium Transport I	1.72	2	0.200	*Atp2a2, Atp2b2*
9	STAT3 Pathway	1.63	5	0.068	*Map3k9, Pias3, Mapk10, Fgfr2, Ddr1*
10	Amyloid Processing	1.60	4	0.078	*Capn5, Bace1, Bace2, App*
11	CD27 Signaling in Lymphocytes	1.54	4	0.076	*Map3k9, Cd70, Mapk10, Cycs*
12	3-phosphoinositide Biosynthesis	1.48	9	0.045	*Plpp6, Wbp11, Nudt16, Ppfibp2, Ppp1r14a, Fgfr2, Erbb3, Mtmr7, Pip4k2a*
13	Superpathway of Inositol Phosphate Compounds	1.45	10	0.043	*Plpp6, Wbp11, Nudt16, Ppfibp2, Ppp1r14a, Fgfr2, Erbb3, Inppl1, Mtmr7, Pip4k2a*
14	Mitochondrial Dysfunction	1.45	8	0.047	*Pdha1, Cox17, Cox6b2, Mapk10, Bace1, Cycs, Bace2, App*
15	TWEAK Signaling	1.39	3	0.086	*Cycs, Bag4, Traf1*
16	Axonal Guidance Signaling	1.37	16	0.035	*Efna2, Pdgfa, Abl1, Fgfr2, Gnaz, Bcar1, Prkcg, Tuba8, Mag, Adam23, Ablim2, Efnb3, Plcl1, Plxnb3, Shank2, Adamts4*
17	UDP-D-xylose and UDP-D-glucuronate Biosynthesis	1.36	1	0.500	*Uxs1*
18	L-glutamine Biosynthesis II (tRNA-dependent)	1.36	1	0.500	*Qrsl1*
19	Leukocyte Extravasation Signaling	1.36	9	0.043	*Cldn11, Edil3, Mapk10, Abl1, Thy1, Fgfr2, Cldn14, Bcar1, Prkcg*
20	Induction of Apoptosis by HIV1	1.35	4	0.066	*Mapk10, Slc25a3, Cycs, Traf1*
21	Parkinson's Signaling	1.33	2	0.125	*Gpr37, Cycs*

B

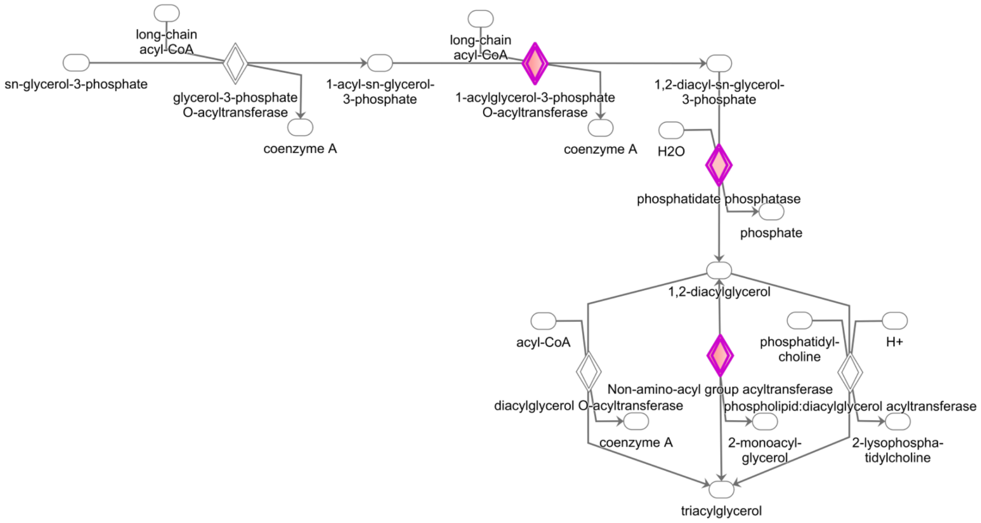

Supplemental Figure 4. Canonical pathway and network analysis of genes within the NPC1-dependent-8 module. (**A**) All significant canonical pathways, as identified by IPA, for the genes of the NPC1-dependent-8 module. (**B**) The IPA canonical pathway for Triacylglycerol Biosynthesis. Genes included in the NPC1-dependent-8 module are highlighted in pink.

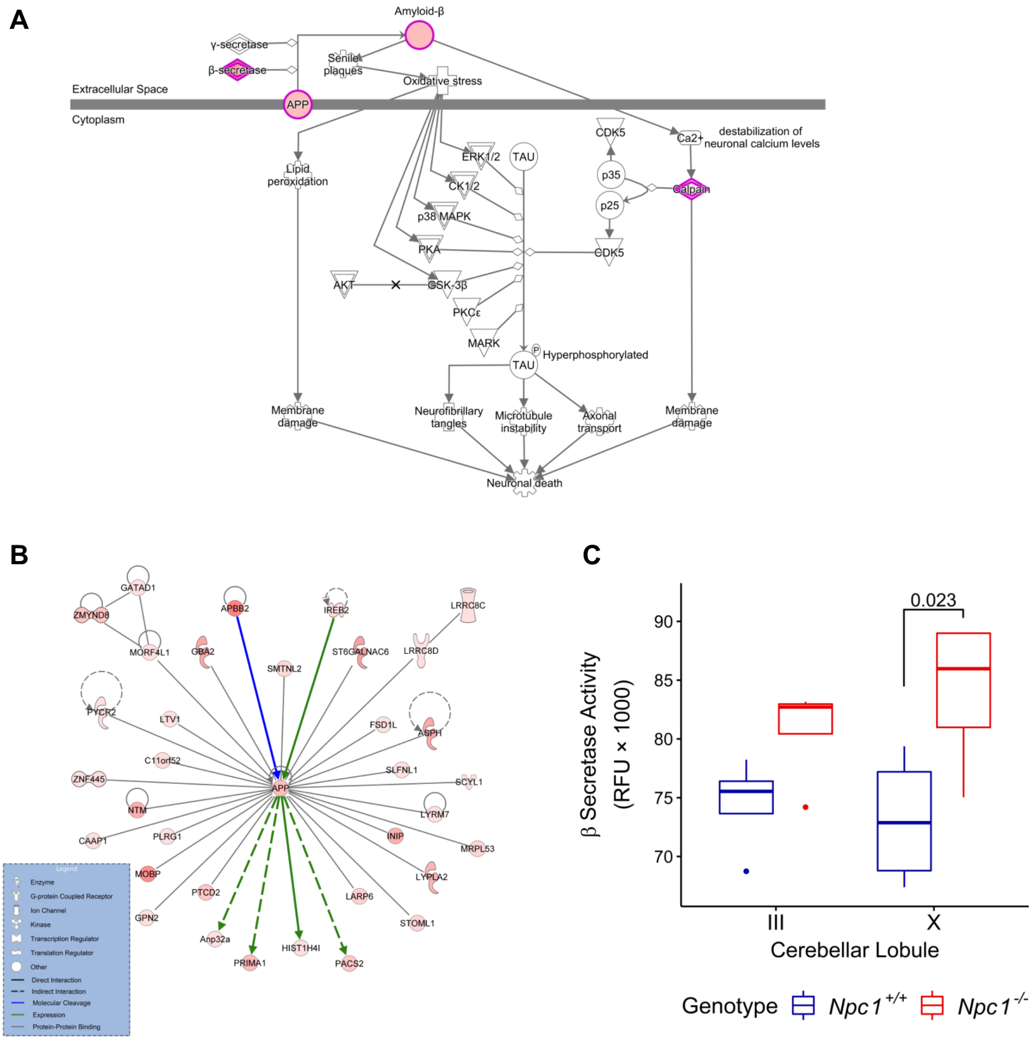

Supplemental Figure 5. Amyloid processing is altered across the *Npc1*-/- cerebellum. (**A**) The IPA canonical pathway for Amyloid Signaling, with genes included in the NPC1-dependent-8 module highlighted in pink. (**B**) The IPA generated network with the centralized *App* gene. All pictured genes are included in the NPC1-dependent-8 module, with the intensity of red color indicating the correlation of each gene to the NPC1-dependent-8 module eigengene. Solid lines indicate direct interactions, while dotted lines indicate indirect interactions between genes. Blue lines indicate molecular cleavage, green lines indicate activation of expression, and gray lines indicated protein-protein binding. (**C**) β-Secretase activity measured across the cerebellar lobules of the *Npc1*+/+ (blue) or *Npc1*-/- (red) mice.

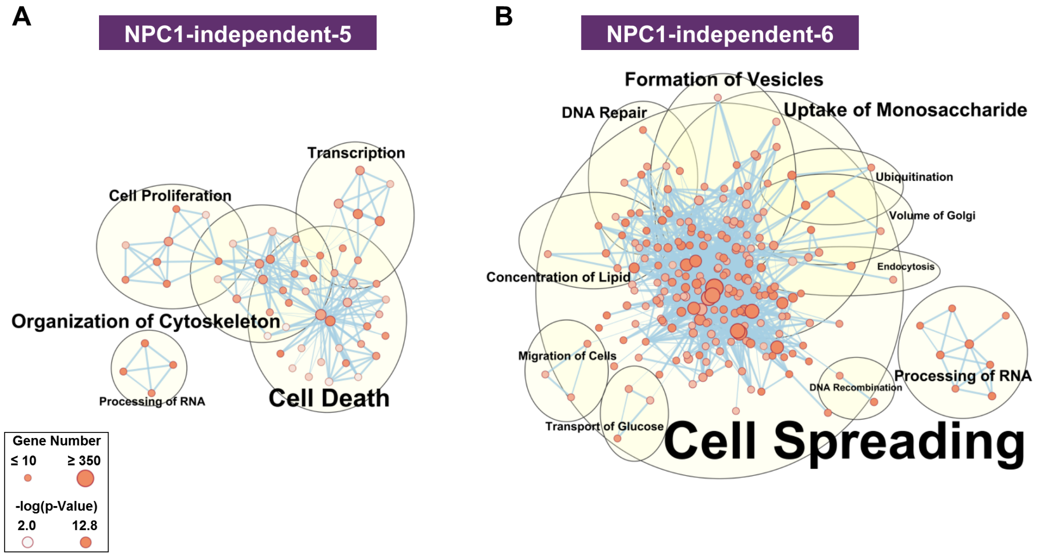

Supplemental Figure 6. Functional enrichment for additional NPC1-independent expression pattern modules. IPA functional analysis of the significantly enriched molecular and cellular functions for the NPC1-independent-5 (**A**) and NPC1-independent-6 (**B**) modules.

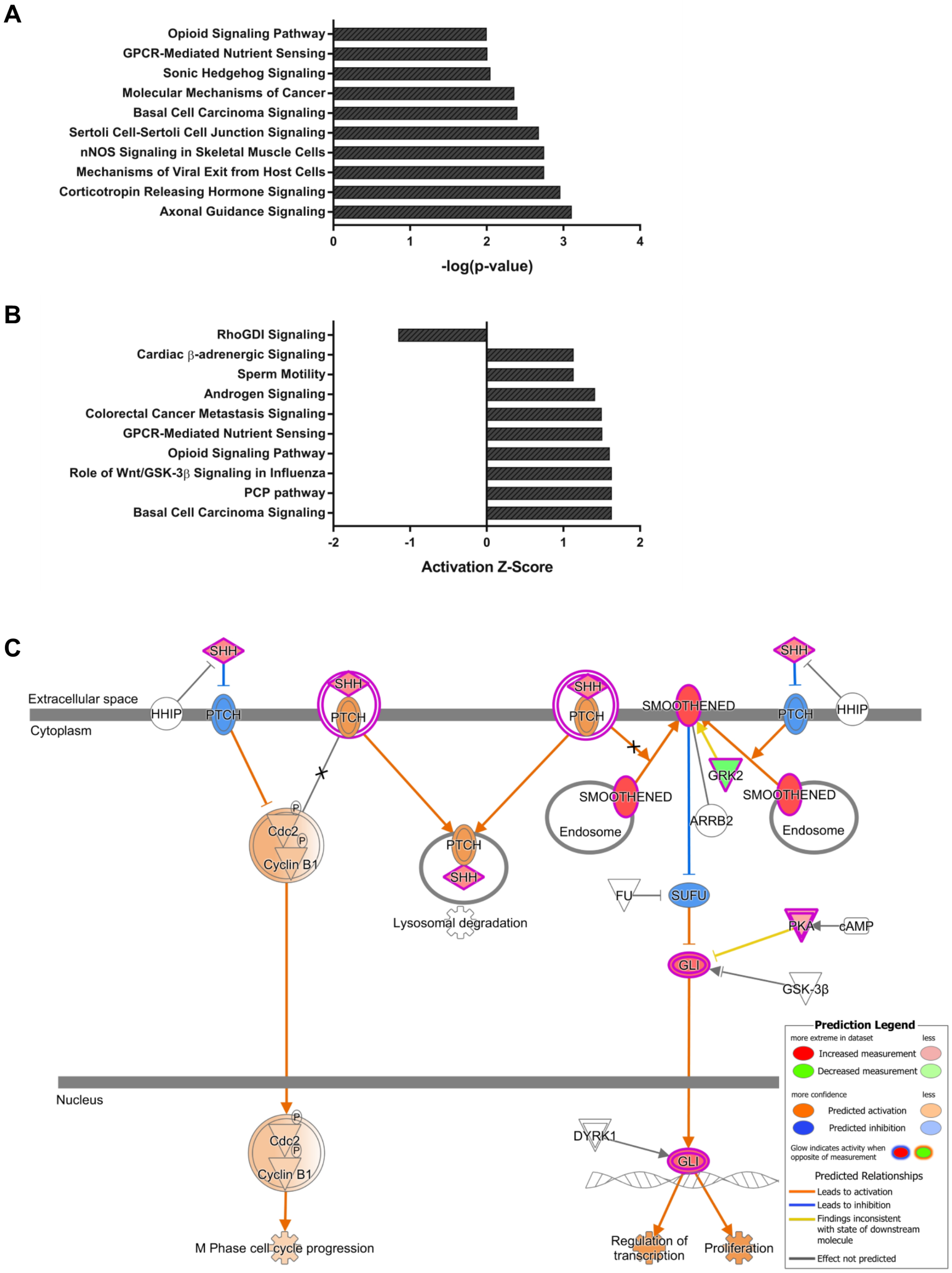

Supplemental Figure 7. IPA canonical pathway analysis for the combined NPC1-independent-3 and 4 modules. (**A**) The top ten most significant canonical IPA pathways for the combined NPC1-independent-3 and 4 modules. (**B**) The most significant IPA canonical pathways with Z-scores greater than 1.5 or less than -1.5. (**C**) The IPA canonical pathway for Sonic Hedgehog Signaling. Genes colored in red have increased expression in lobule X and those colored in green have decreased expression in lobule X, compared to the anterior lobules. The IPA generated molecular activity predictions are also depicted, with activation in lobule X depicted as orange lines and inhibition in lobule X depicted as blue lines.

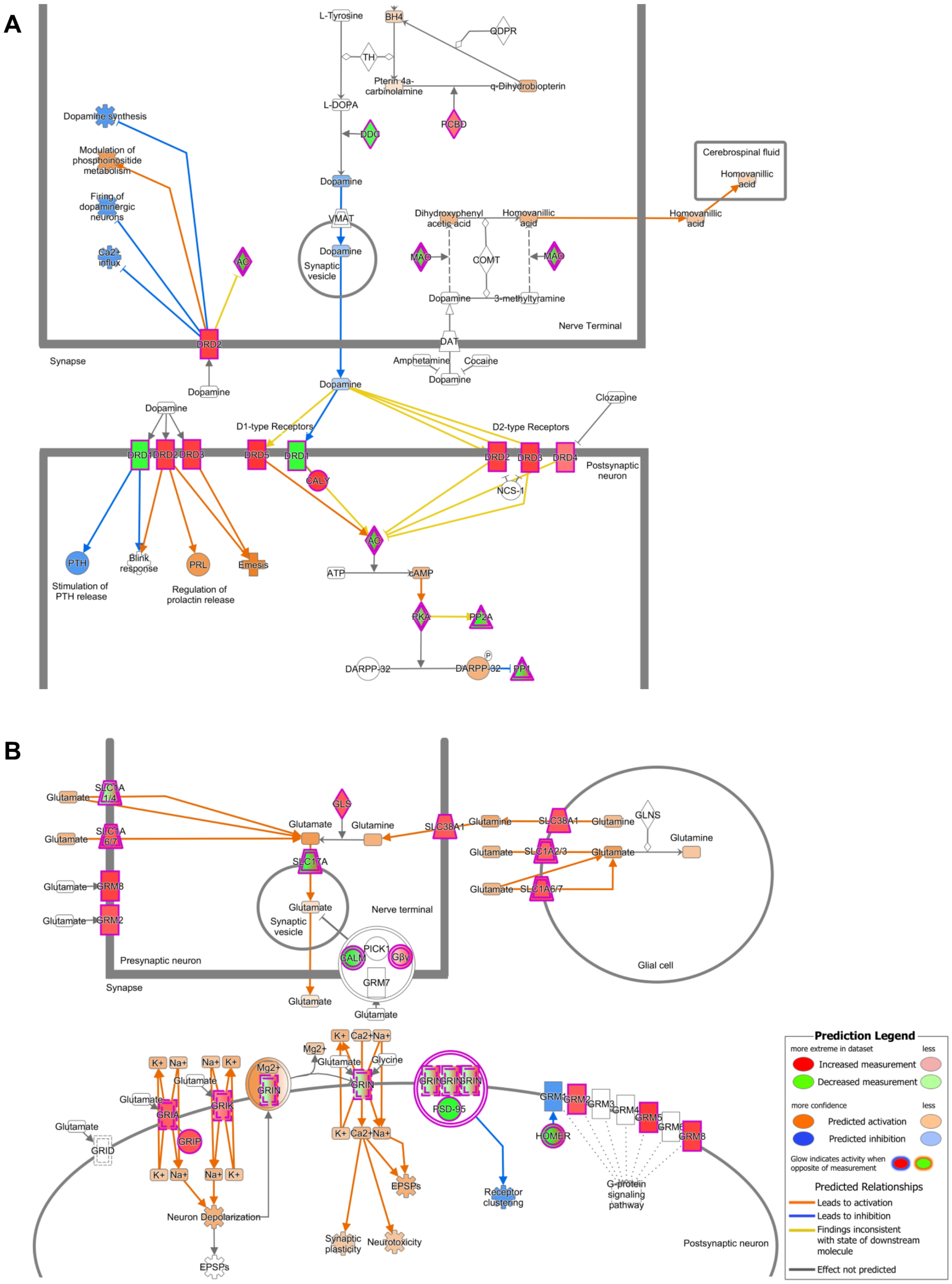

Supplemental Figure 8. IPA canonical pathway analysis for neurotransmitter receptor signaling in the combined NPC1-independent-1 and 2 modules. (**A**) The IPA canonical pathway for Dopamine Receptor Signaling. Genes colored in red have increased expression in lobule X and those colored in green have decreased expression in lobule X, compared to the anterior lobules. The IPA generated molecular activity predictions are also depicted, with activation in lobule X depicted as orange lines and inhibition in lobule X depicted as blue lines. (**B**) The IPA canonical pathway for Glutamate Receptor Signaling.

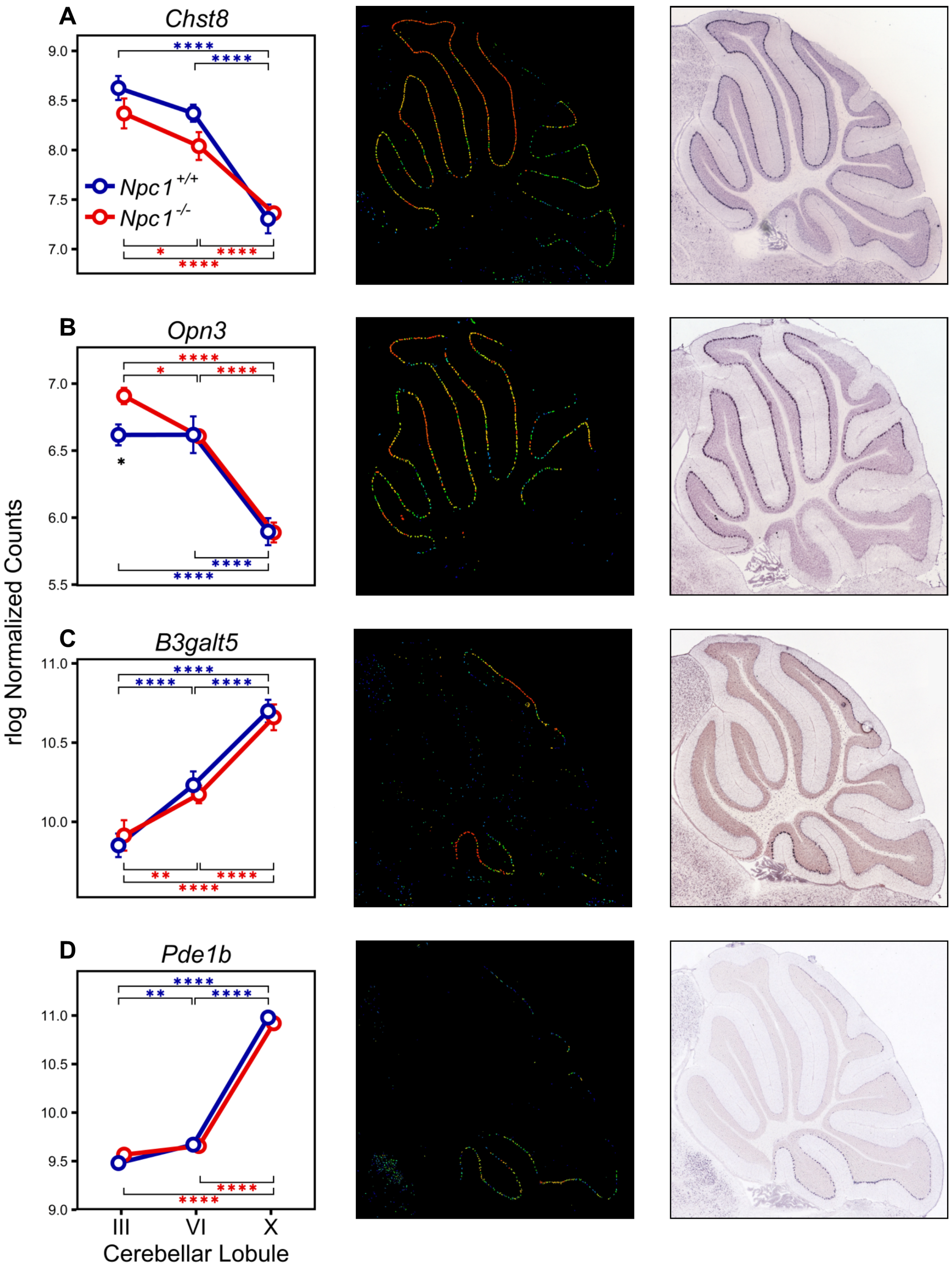

Supplemental Figure 9. Validation of Purkinje neuron specific gene expression. Four genes that were previously identified to be Purkinje neuron specific and having differential anterior-to-posterior cerebellar gene expression [88] are shown. Examples of two genes previously noted with decreasing gene expression, *Chst8* (**A**) and *Opn3* (**B**), are shown. The left panel of each shows the expression of each gene across lobules III, VI, and X from our RNA-sequencing experiment. The middle panel shows color coded gene expression from the Allen Brain Atlas, with red representing high expression, blue showing low expression, and black depicting a lack of expression. The right panel is the ISH image from the Allen Brain Atlas, showing all ten lobules of the entire cerebellum. Examples of two genes previously identified as Purkinje neuron specific with increased gene expression, *B3galt5* (**C**) and *Pde1b* (**D**).

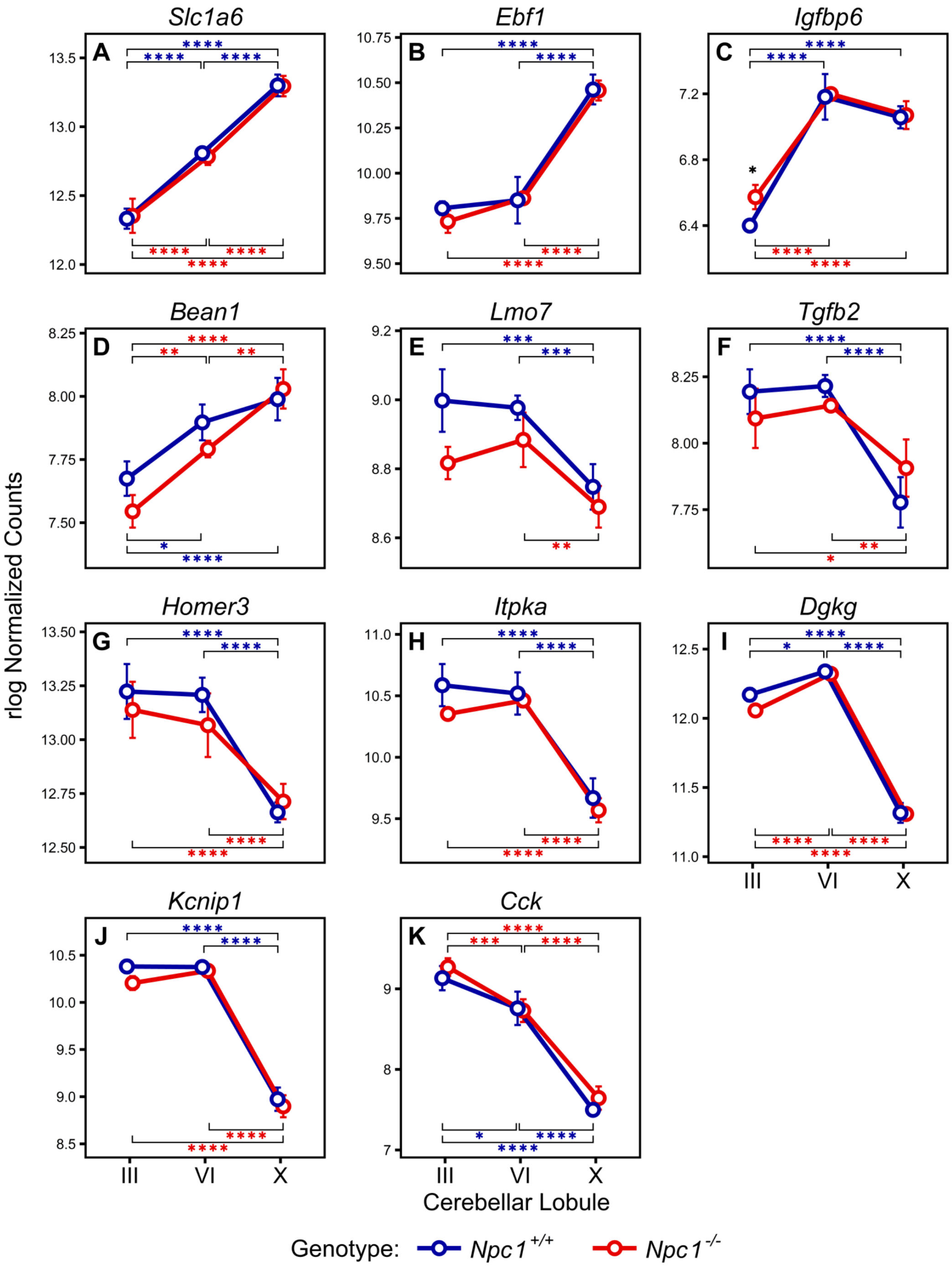

Supplemental Figure 10. Purkinje neuron specific genes identified as having increased or decreased expression pattern across the lobules. (**A-D**) Four genes previously verified to be solely expressed in Purkinje neurons that have been identified as having increased gene expression across the lobules via our RNA-sequencing data set in both *Npc1*$^{+/+}$ and *Npc1*$^{-/-}$ samples. (**E-K**) Seven Purkinje neuron enriched genes that were found to have decreased gene expression across the lobules.

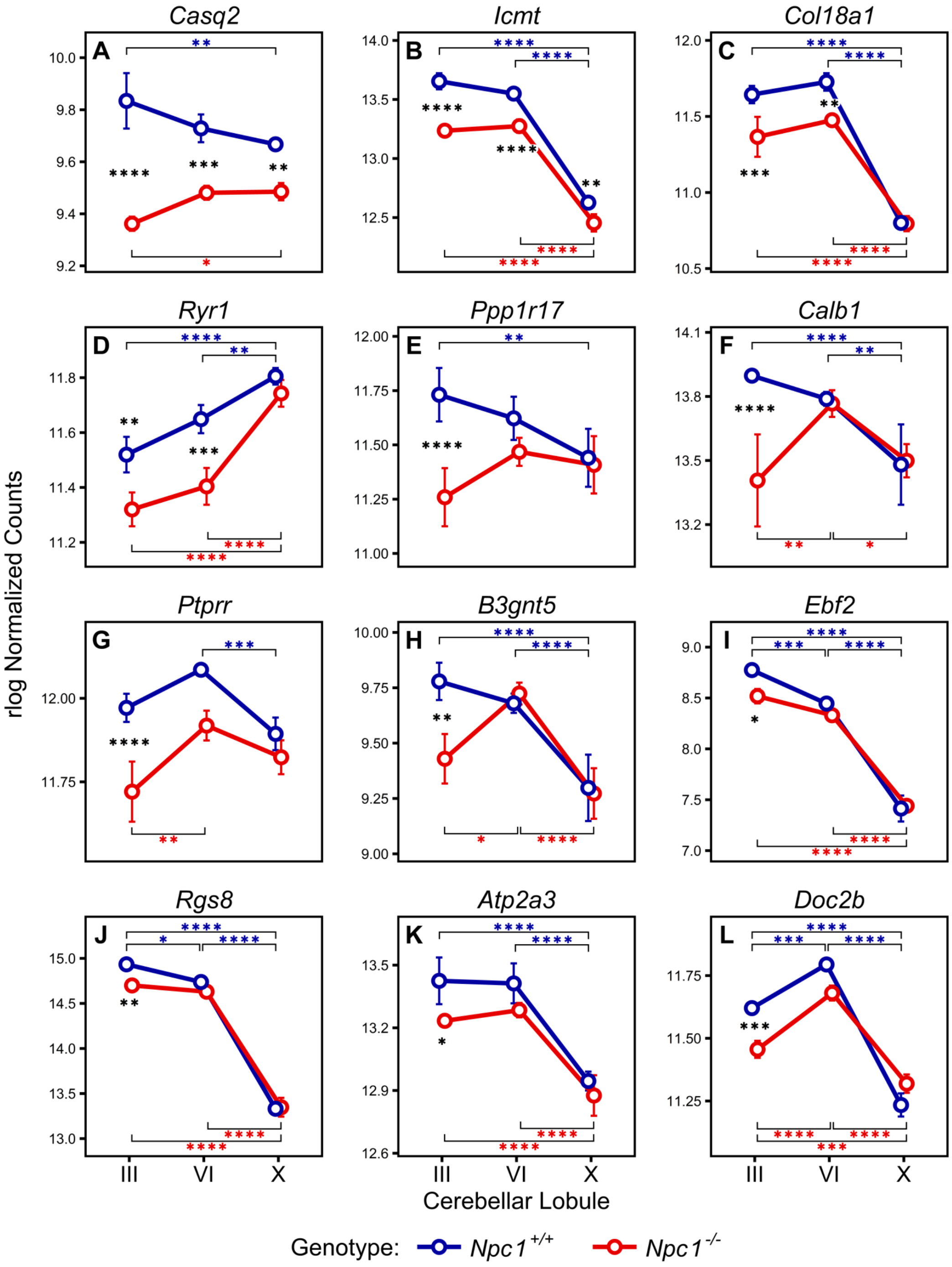

Supplemental Figure 11. Purkinje neuron specific genes identified as having decreased expression across the *Npc1*$^{+/+}$ lobules and decreased expression in *Npc1*$^{-/-}$ lobules when compared to *Npc1*$^{+/+}$ lobules. (**A-B**) The two genes previously verified to be solely expressed in Purkinje neurons that have decreased expression in all *Npc1*$^{-/-}$ lobules and decreased expression across the lobules of *Npc1*$^{+/+}$ samples. (**C-D**) The two Purkinje neuron enriched genes that have significantly decreased expression in lobules III and VI in *Npc1*$^{-/-}$ samples and decreased or increased expression across the lobules of *Npc1*$^{+/+}$ samples, respectively. (**E-L**) Eight Purkinje neuron enriched genes that have decreased expression in lobule III of *Npc1*$^{-/-}$ samples and decreased expression across the lobules of *Npc1*$^{+/+}$ samples.

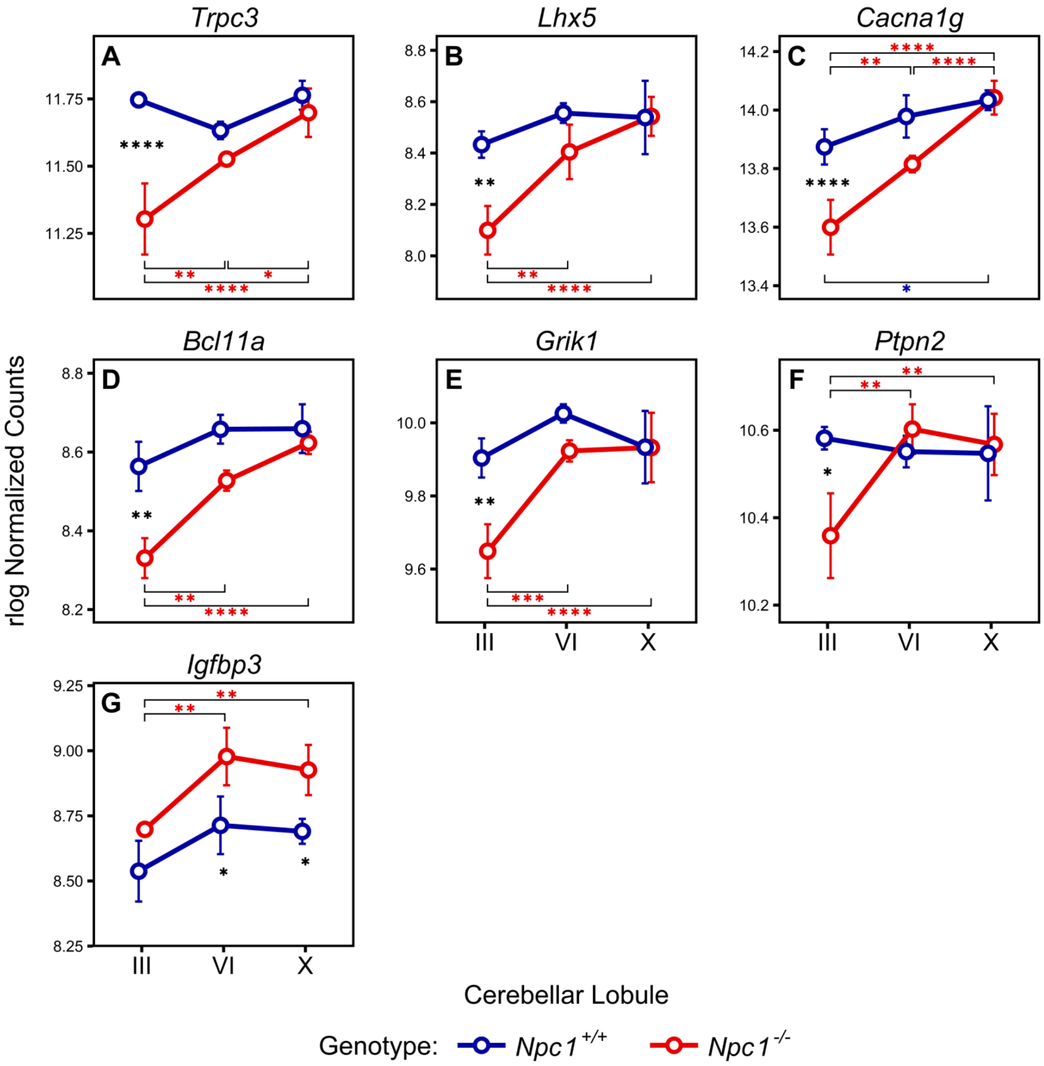

Supplemental Figure 12. Purkinje neuron specific genes that have no change in gene expression within *Npc1*$^{+/+}$ lobules and decreased expression in *Npc1*$^{-/-}$ lobules when compared to *Npc1*$^{+/+}$ lobules. (**A-F**) The six genes previously verified to be solely expressed in Purkinje neurons that have decreased expression in lobule III of *Npc1*$^{-/-}$ samples and no change in expression across the lobules of *Npc1*$^{+/+}$ samples. (**G**) One Purkinje neuron enriched gene that has no change in expression across the *Npc1*$^{+/+}$ lobules and increased expression in lobules VI and X of the *Npc1*$^{-/-}$ samples.

Supplemental Table 1. Significantly up/downregulated genes in lobules III, VI, and X between *Npc1*$^{+/+}$ and *Npc1*$^{-/-}$ cerebella

Rank	Gene	Gene Name	Average log_2FC	Average $-log_{10}pAdj$
**Significantly increased genes in *Npc1*$^{-/-}$ lobules III, VI, and X when compared to *Npc1*$^{+/+}$ lobules III, VI, and X, respectively**				
1	*Lyz2*	lysozyme 2	3.29	40.76
2	*Mpeg1*	macrophage expressed gene 1	2.59	30.34
3	*Gpnmb*	glycoprotein (transmembrane) nmb	3.40	22.53
4	*C4b*	complement component 4B (Chido blood group)	2.24	20.16
5	*C1qa*	complement component 1, q subcomponent, alpha polypeptide	1.76	12.90
6	*C1qb*	complement component 1, q subcomponent, beta polypeptide	1.80	9.65
7	*Tyrobp*	TYRO protein tyrosine kinase binding protein	2.19	8.89
8	*Ctss*	cathepsin S	1.25	8.89
9	*Mmp12*	matrix metallopeptidase 12	7.07	7.94
10	*Stab1*	stabilin 1	0.96	7.92
11	*Trem2*	triggering receptor expressed on myeloid cells 2	2.04	7.42
12	*C1qc*	complement component 1, q subcomponent, C chain	1.93	7.16
13	*Ctsd*	cathepsin D	0.68	6.75
14	*Itgax*	integrin alpha X	3.00	6.53
15	*C3ar1*	complement component 3a receptor 1	2.49	6.42
16	*Clec7a*	C-type lectin domain family 7, member a	3.44	5.55
17	*Fcrls*	Fc receptor-like S, scavenger receptor	2.14	5.46
18	*Hexb*	hexosaminidase B	0.79	5.44
19	*Icam1*	intercellular adhesion molecule 1	1.50	5.35
20	*Cd84*	CD84 antigen	1.82	5.02
21	*Myo1f*	myosin IF	1.54	4.36
22	*Ly9*	lymphocyte antigen 9	3.62	4.29
23	*Acer2*	alkaline ceramidase 2	0.94	4.23
24	*Cybb*	cytochrome b-245, beta polypeptide	2.03	4.09
25	*B2m*	beta-2 microglobulin	0.91	4.03
26	*Xdh*	xanthine dehydrogenase	1.13	3.96
27	*Adgre1*	adhesion G protein-coupled receptor E1	1.93	3.96
28	*Cd22*	CD22 antigen	5.10	3.87
29	*Timp3*	tissue inhibitor of metalloproteinase 3	0.46	3.68
30	*Hpse*	heparanase	3.86	3.54
31	*Apobec1*	apolipoprotein B mRNA editing enzyme, catalytic polypeptide 1	1.85	3.36
32	*Ptgds*	prostaglandin D2 synthase (brain)	0.78	3.35
33	*Lgals3*	lectin, galactose binding, soluble 3	1.72	3.35
34	*Cd68*	CD68 antigen	1.69	3.29
35	*Rhobtb2*	Rho-related BTB domain containing 2	0.42	3.12
36	*Hvcn1*	hydrogen voltage-gated channel 1	1.70	3.12
37	*Nckap1l*	NCK associated protein 1 like	0.96	3.09
38	*Lilrb4a*	leukocyte immunoglobulin-like receptor, subfamily B, member 4A	3.05	3.01
39	*Itgb2*	integrin beta 2	1.49	2.77
40	*C2*	complement component 2 (within H-2S)	0.97	2.65
41	*Aspg*	asparaginase	1.32	2.57
42	*Lgals3bp*	lectin, galactoside-binding, soluble, 3 binding protein	1.14	2.53
43	*Parp14*	poly (ADP-ribose) polymerase family, member 14	0.64	2.30
44	*Rnase4*	ribonuclease, RNase A family 4	1.05	2.30
45	*Atp6v0d2*	ATPase, H+ transporting, lysosomal V0 subunit D2	4.81	2.21
46	*Laptm5*	lysosomal-associated protein transmembrane 5	0.98	2.13
47	*Cd36*	CD36 molecule	2.05	2.11
48	*Vwf*	Von Willebrand factor	0.45	2.10
49	*Apod*	apolipoprotein D	0.56	2.03
50	*Cd93*	CD93 antigen	0.43	2.03
51	*Csf1r*	colony stimulating factor 1 receptor	0.61	2.01
52	*Siglec1*	sialic acid binding Ig-like lectin 1, sialoadhesin	0.85	1.98

53	*Tlr13*	toll-like receptor 13	1.51	1.93
54	*Tlr2*	toll-like receptor 2	1.49	1.89
55	*Dcn*	decorin	0.51	1.86
56	*Osmr*	oncostatin M receptor	0.44	1.84
57	*Prodh*	proline dehydrogenase	0.44	1.81
58	*Apcdd1*	adenomatosis polyposis coli down-regulated 1	0.31	1.80
59	*Ms4a7*	membrane-spanning 4-domains, subfamily A, member 7	1.19	1.57
60	*Slfn5*	schlafen 5	0.44	1.56
**Significantly decreased genes in *Npc1*$^{-/-}$ lobules III, VI, and X when compared to *Npc1*$^{+/+}$ lobules III, VI, and X, respectively**				
1	*Npc1*	NPC intracellular cholesterol transporter 1	-1.47	73.02
2	*Icmt*	isoprenylcysteine carboxyl methyltransferase	-0.39	2.78
3	*Casq2*	calsequestrin 2	-0.45	2.49
4	*Psd2*	pleckstrin and Sec7 domain containing 2	-0.22	1.79
5	*Hhip*	Hedgehog-interacting protein	-0.45	1.79
6	*Gpr63*	G protein-coupled receptor 63	-0.48	1.77

Supplemental Table 2. Significantly up/downregulated genes in lobules III and VI between *Npc1*$^{+/+}$ and *Npc1*$^{-/-}$ cerebella

Rank	Gene	Gene Name	Average $\log_2$FC	Average $-\log_{10}$pAdj
**Significantly increased genes in *Npc1*$^{-/-}$ lobules III and VI when compared to *Npc1*$^{+/+}$ lobules III and VI, respectively**				
1	*Olfml3*	olfactomedin-like 3	0.73	3.03
2	*Th*	tyrosine hydroxylase	2.82	2.41
3	*Lcp1*	lymphocyte cytosolic protein 1	1.03	2.04
4	*Rtel1*	regulator of telomere elongation helicase 1	0.40	1.98
5	*Ctsb*	cathepsin B	0.26	1.93
6	*D130043K22Rik*	RIKEN cDNA D130043K22 gene	0.42	1.87
7	*Pros1*	protein S (alpha)	0.56	1.80
8	*Ptgfrn*	prostaglandin F2 receptor negative regulator	0.46	1.78
9	*Aif1*	allograft inflammatory factor 1	1.33	1.72
10	*Plin2*	perilipin 2	0.44	1.62
11	*Parp9*	poly (ADP-ribose) polymerase family, member 9	0.50	1.59
**Significantly decreased genes in *Npc1*$^{-/-}$ lobules III and VI when compared to *Npc1*$^{+/+}$ lobules III and VI, respectively**				
1	*Prkcg*	protein kinase C, gamma	-0.42	6.36
2	*Tspan2*	tetraspanin 2	-0.47	4.49
3	*Camk2a*	calcium/calmodulin-dependent protein kinase II alpha	-0.40	3.39
4	*Nell1*	NEL-like 1	-0.62	3.29
5	*Ryr1*	ryanodine receptor 1, skeletal muscle	-0.31	2.88
6	*Garnl3*	GTPase activating RANGAP domain-like 3	-0.32	2.82
7	*Col18a1*	collagen, type XVIII, alpha 1	-0.34	2.82
8	*Hapln4*	hyaluronan and proteoglycan link protein 4	-0.28	2.31
9	*Trabd2b*	TraB domain containing 2B	-0.40	2.23
10	*Strip2*	striatin interacting protein 2	-0.28	2.16
11	*Gabbr1*	gamma-aminobutyric acid (GABA) B receptor, 1	-0.22	2.09
12	*Scn4b*	sodium channel, type IV, beta	-0.65	1.99
13	*Faap100*	Fanconi anemia core complex associated protein 100	-0.32	1.76
14	*Slc9a3*	solute carrier family 9 (sodium/hydrogen exchanger), member 3	-0.42	1.71
15	*Far2*	fatty acyl CoA reductase 2	-0.56	1.64
16	*Nek2*	NIMA (never in mitosis gene a)-related expressed kinase 2	-0.31	1.62
17	*Gask1b*	golgi associated kinase 1B	-0.61	1.57
18	*Grm3*	glutamate receptor, metabotropic 3	-0.21	1.54
20	*Arap1*	ArfGAP with RhoGAP domain, ankyrin repeat and PH domain 1	-0.20	1.50
21	*Svep1*	sushi, von Willebrand factor type A, EGF and pentraxin domain containing 1	-0.20	1.39
**Genes significantly changed in opposite directions in *Npc1*$^{-/-}$ lobules III and VI when compared to *Npc1*$^{+/+}$ lobules III and VI**				
1	*AW146154*	expressed sequence AW146154	-0.02	1.54

Supplemental Table 3. Significantly up/downregulated genes exclusively in lobule X between *Npc1*$^{+/+}$ and *Npc1*$^{-/-}$ cerebella

Rank	Gene	Gene Name	log_2FC	$-log_{10}pAdj$
**Significantly increased genes exclusively in *Npc1*$^{-/-}$ lobule X when compared to *Npc1*$^{+/+}$ lobule X**				
1	*Thbs1*	thrombospondin 1	0.89	6.24
2	*Myof*	myoferlin	0.64	4.39
3	*Nrp1*	neuropilin 1	0.37	2.89
4	*Grid2ip*	glutamate receptor, ionotropic, delta 2 (Grid2) interacting protein 1	0.32	2.55
5	*Npr3*	natriuretic peptide receptor 3	1.66	2.52
6	*Igf1*	insulin-like growth factor 1	0.62	2.31
7	*Kdr*	kinase insert domain protein receptor	0.29	2.18
8	*Sema3b*	sema domain, immunoglobulin domain (Ig), short basic domain, secreted, (semaphorin) 3B	0.85	2.17
9	*Mlph*	melanophilin	0.53	2.15
10	*Abcc9*	ATP-binding cassette, sub-family C (CFTR/MRP), member 9	0.37	2.11
11	*P4ha1*	procollagen-proline, 2-oxoglutarate 4-dioxygenase (proline 4-hydroxylase), alpha 1 polypeptide	0.25	2.07
12	*Cnr2*	cannabinoid receptor 2 (macrophage)	2.55	2.04
13	*Wnk4*	WNK lysine deficient protein kinase 4	0.73	2.04
14	*Icosl*	icos ligand	0.36	1.99
15	*Postn*	periostin, osteoblast specific factor	1.39	1.94
16	*Phyhd1*	phytanoyl-CoA dioxygenase domain containing 1	0.39	1.93
17	*Acacb*	acetyl-Coenzyme A carboxylase beta	0.29	1.88
18	*Myo1e*	myosin IE	0.26	1.84
19	*Ttr*	transthyretin	2.41	1.84
20	*Igfn1*	immunoglobulin-like and fibronectin type III domain containing 1	2.08	1.83
21	*Kl*	klotho	1.72	1.77
22	*Trpv4*	transient receptor potential cation channel, subfamily V, member 4	1.51	1.77
23	*Rad50*	RAD50 double strand break repair protein	0.24	1.70
24	*Lmo2*	LIM domain only 2	0.32	1.67
25	*Abca4*	ATP-binding cassette, sub-family A (ABC1), member 4	0.86	1.65
26	*Gucy2f*	guanylate cyclase 2f	1.81	1.65
27	*F5*	coagulation factor V	1.04	1.52
28	*Slc19a3*	solute carrier family 19, member 3	0.41	1.48
29	*Rab3il1*	RAB3A interacting protein (rabin3)-like 1	0.48	1.45
30	*Emilin1*	elastin microfibril interfacer 1	0.53	1.39
31	*Rasgrp1*	RAS guanyl releasing protein 1	0.22	1.39
32	*Trim25*	tripartite motif-containing 25	0.39	1.38
33	*Grasp*	GRP1 (general receptor for phosphoinositides 1)-associated scaffold protein	0.54	1.32
34	*Itpripl1*	inositol 1,4,5-triphosphate receptor interacting protein-like 1	0.59	1.32
35	*Ptpn14*	protein tyrosine phosphatase, non-receptor type 14	0.27	1.32
36	*B3glct*	beta-3-glucosyltransferase	0.23	1.30
**Significantly decreased genes exclusively in *Npc1*$^{-/-}$ lobule X when compared to *Npc1*$^{+/+}$ lobule X**				
1	*Cd163*	CD163 antigen	-1.68	2.49
2	*Rasgrp2*	RAS, guanyl releasing protein 2	-0.43	1.88
3	*Cdh4*	cadherin 4	-0.22	1.46
4	*Adam23*	a disintegrin and metallopeptidase domain 23	-0.22	1.37
